# Impact of respiratory bacterial infections on mortality in Japanese patients with COVID-19: a retrospective cohort study

**DOI:** 10.1186/s12890-023-02418-3

**Published:** 2023-04-26

**Authors:** Kensuke Nakagawara, Hirofumi Kamata, Shotaro Chubachi, Ho Namkoong, Hiromu Tanaka, Ho Lee, Shiro Otake, Takahiro Fukushima, Tatsuya Kusumoto, Atsuho Morita, Shuhei Azekawa, Mayuko Watase, Takanori Asakura, Katsunori Masaki, Makoto Ishii, Akifumi Endo, Ryuji Koike, Hiroyasu Ishikura, Tohru Takata, Yasushi Matsushita, Norihiro Harada, Hiroyuki Kokutou, Takashi Yoshiyama, Kensuke Kataoka, Yoshikazu Mutoh, Masayoshi Miyawaki, Soichiro Ueda, Hiroshi Ono, Takuya Ono, Tomohisa Shoko, Hiroyuki Muranaka, Kodai Kawamura, Nobuaki Mori, Takao Mochimaru, Mototaka Fukui, Yusuke Chihara, Yoji Nagasaki, Masaki Okamoto, Masaru Amishima, Toshio Odani, Mayuko Tani, Koichi Nishi, Yuya Shirai, Ryuya Edahiro, Akira Ando, Naozumi Hashimoto, Shinji Ogura, Yuichiro Kitagawa, Toshiyuki Kita, Takashi Kagaya, Yasuhiro Kimura, Naoki Miyazawa, Tomoya Tsuchida, Shigeki Fujitani, Koji Murakami, Hirohito Sano, Yuki Sato, Yoshinori Tanino, Ryo Otsuki, Shuko Mashimo, Mizuki Kuramochi, Yasuo Hosoda, Yoshinori Hasegawa, Tetsuya Ueda, Yotaro Takaku, Takashi Ishiguro, Akiko Fujiwara, Naota Kuwahara, Hideya Kitamura, Eri Hagiwara, Yasushi Nakamori, Fukuki Saito, Yuta Kono, Shinji Abe, Tomoo Ishii, Takehiko Ohba, Yu Kusaka, Hiroko Watanabe, Makoto Masuda, Hiroki Watanabe, Yoshifumi Kimizuka, Akihiko Kawana, Yu Kasamatsu, Satoru Hashimoto, Yukinori Okada, Tomomi Takano, Kazuhiko Katayama, Masumi Ai, Atsushi Kumanogoh, Toshiro Sato, Katsushi Tokunaga, Seiya Imoto, Yuko Kitagawa, Akinori Kimura, Satoru Miyano, Naoki Hasegawa, Seishi Ogawa, Takanori Kanai, Koichi Fukunaga, The Japan COVID-19 Task Force

**Affiliations:** 1 Division of Pulmonary Medicine, Department of Medicine, Keio University School of Medicine, 35 Shinanomachi, Shinjuku-Ku, Tokyo, 160-8582 Japan; 2 Department of Infectious Diseases, Keio University School of Medicine, Tokyo, Japan; 3 Department of Clinical Medicine (Laboratory of Bioregulatory Medicine), Kitasato University School of Pharmacy, Tokyo, Japan; 4 Department of Respiratory Medicine, Kitasato University, Kitasato Institute Hospital, Tokyo, Japan; 5 Department of Respiratory Medicine, Nagoya University Graduate School of Medicine, Nagoya, Japan; 6 Clinical Research Center, Tokyo Medical and Dental University Hospital of Medicine, Tokyo, Japan; 7 Department of Emergency and Critical Care Medicine, Faculty of Medicine, Fukuoka University Hospital, Fukuoka, Japan; 8 Department of Infection Control, Fukuoka University, Fukuoka, Japan; 9 Department of Internal Medicine and Rheumatology, Faculty of Medicine and Graduate School of Medicine, Juntendo University, Tokyo, Japan; 10 Department of Respiratory Medicine, Faculty of Medicine and Graduate School of Medicine, Juntendo University, Tokyo, Japan; 11 Fukujuji Hospital, Kiyose, Japan; 12 Department of Respiratory Medicine and Allergy, Tosei General Hospital, Seto, Japan; 13 Department of Infectious Diseases, Tosei General Hospital, Seto, Japan; 14 Department of Internal Medicine, JCHO (Japan Community Health Care Organization, Saitama Medical Center, Saitama, Japan; 15 Division of Infectious Diseases and Respiratory Medicine, Kumamoto Medical Center, Kumamoto, Japan; 16 Emergency and Critical Care Medicine, Tokyo Women’s Medical University Adachi Medical Center, Tokyo, Japan; 17 Division of Respiratory Medicine, Social Welfare Organization Saiseikai Imperial Gift Foundation, Inc, Saiseikai Kumamoto Hospital, Kumamoto, Japan; 18 Department of General Internal Medicine and Infectious Diseases, National Hospital Organization Tokyo Medical Center, Tokyo, Japan; 19 Department of Respiratory Medicine, National Hospital Organization Tokyo Medical Center, Tokyo, Japan; 20 Uji-Tokushukai Medical Center, Uji, Japan; 21 Department of Respirology, National Hospital Organization Kyushu Medical Center, Fukuoka, Japan; 22 Department of Respiratory Medicine, National Hospital Organization Hokkaido Medical Center, Sapporo, Japan; 23 Department of Rheumatology, National Hospital Organization Hokkaido Medical Center, Sapporo, Japan; 24 Ishikawa Prefectural Central Hospital, Kanazawa, Japan; 25 Department of Respiratory Medicine and Clinical Immunology, Osaka University Graduate School of Medicine, Osaka, Japan; 26 Department of Emergency and Disaster Medicine, Gifu University Graduate School of Medicine, Gifu, Japan; 27 National Hospital Organization Kanazawa Medical Center, Kanazawa, Japan; 28 Department of Respiratory Medicine, Saiseikai Yokohamashi Nanbu Hospital, Yokohama, Japan; 29 Division of General Internal Medicine, Department of Internal Medicine, St. Marianna University School of Medicine Kawasaki, Kawasaki, Japan; 30 Department of Emergency and Critical Care Medicine, St. Marianna University School of Medicine, Kawasaki, Japan; 31 Department of Respiratory Medicine, Tohoku University Graduate School of Medicine, Sendai, Japan; 32 Department of Pulmonary Medicine, School of Medicine, Fukushima Medical University, Fukushima, Japan; 33 Department of Respiratory Medicine, Toyohashi Municipal Hospital, Toyohashi, Japan; 34 Department of Gastroenterology, National Hospital Organization Saitama National Hospital, Wako, Saitama Japan; 35 Department of Respiratory Medicine, Osaka Saiseikai Nakatsu Hospital, Osaka, Japan; 36 Department of Respiratory Medicine, Saitama Cardiovascular and Respiratory Center, Kumagaya, Japan; 37 Department of Medicine, Division of Allergology and Respiratory Medicine, Showa University Koto Toyosu Hospital, Tokyo, Japan; 38 Department of Respiratory Medicine, Kanagawa Cardiovascular and Respiratory Center, Yokohama, Japan; 39 Department of Emergency and Critical Care Medicine, Kansai Medical University General Medical Center, Osaka, Japan; 40 Department of Respiratory Medicine, Tokyo Medical University Hospital, Tokyo, Japan; 41 Tokyo Medical University Ibaraki Medical Center, Inashiki, Japan; 42 Department of Respiratory Medicine, Ome Municipal General Hospital, Ome, Tokyo, Japan; 43 Division of Respiratory Medicine, Tsukuba Kinen General Hospital, Ibaraki, Japan; 44 Department of Respiratory Medicine, Fujisawa City Hospital, Fujisawa, Japan; 45 Division of Infectious Diseases and Respiratory Medicine, Department of Internal Medicine, National Defense Medical College, Saitama, Japan; 46 Department of Infection Control and Laboratory Medicine, Kyoto Prefectural University of Medicine, Kyoto, Japan; 47 Department of Anesthesiology and Intensive Care Medicine, Kyoto Prefectural University of Medicine, Kyoto, Japan; 48 Department of Statistical Genetics, Osaka University Graduate School of Medicine, Osaka, Japan; 49 Department of Genome Informatics, Graduate School of Medicine, the University of Tokyo, Tokyo, 113-0033 Japan; 50 Laboratory for Systems Genetics, RIKEN Center for Integrative Medical Sciences, Kanagawa, 230-0045 Japan; 51 School of Veterinary Medicine, Kitasato University, Towada, Japan; 52 Laboratory of Viral Infection I, Department of Infection Control and Immunology, Ōmura Satoshi Memorial Institute & Graduate School of Infection Control Sciences, Kitasato University, Tokyo, Japan; 53 Department of Insured Medical Care Management, Tokyo Medical and Dental University Hospital of Medicine, Tokyo, Japan; 54 Department of Organoid Medicine, Keio University School of Medicine, Tokyo, Japan; 55 Genome Medical Science Project (Toyama), National Center for Global Health and Medicine, Tokyo, Japan; 56 Division of Health Medical Intelligence, Human Genome Center, the Institute of Medical Science, the University of Tokyo, Tokyo, Japan; 57 Department of Surgery, Keio University School of Medicine, Tokyo, Japan; 58 Institute of Research, Tokyo Medical and Dental University, Tokyo, Japan; 59 M&D Data Science Center, Tokyo Medical and Dental University, Tokyo, Japan; 60 Department of Pathology and Tumor Biology, Kyoto University, Kyoto, Japan; 61 Division of Gastroenterology and Hepatology, Department of Medicine, Keio University School of Medicine, Tokyo, Japan

**Keywords:** SARS-CoV-2 infection, Neutrophil–lymphocyte ratio, Mortality, Invasive mechanical ventilation, Intensive care unit

## Abstract

**Background:**

Although cases of respiratory bacterial infections associated with coronavirus disease 2019 (COVID-19) have often been reported, their impact on the clinical course remains unclear. Herein, we evaluated and analyzed the complication rates of bacterial infections, causative organisms, patient backgrounds, and clinical outcome in Japanese patients with COVID-19.

**Methods:**

We performed a retrospective cohort study that included inpatients with COVID-19 from multiple centers participating in the Japan COVID-19 Taskforce (April 2020 to May 2021) and obtained demographic, epidemiological, and microbiological results and the clinical course and analyzed the cases of COVID-19 complicated by respiratory bacterial infections.

**Results:**

Of the 1,863 patients with COVID-19 included in the analysis, 140 (7.5%) had respiratory bacterial infections. Community-acquired co-infection at COVID-19 diagnosis was uncommon (55/1,863, 3.0%) and was mainly caused by *Staphylococcus aureus*, *Klebsiella pneumoniae* and *Streptococcus pneumoniae.* Hospital-acquired bacterial secondary infections, mostly caused by *Staphylococcus aureus*, *Pseudomonas aeruginosa*, and *Stenotrophomonas maltophilia*, were diagnosed in 86 patients (4.6%). Severity-associated comorbidities were frequently observed in hospital-acquired secondary infection cases, including hypertension, diabetes, and chronic kidney disease. The study results suggest that the neutrophil–lymphocyte ratio (> 5.28) may be useful in diagnosing complications of respiratory bacterial infections. COVID-19 patients with community-acquired or hospital-acquired secondary infections had significantly increased mortality.

**Conclusions:**

Respiratory bacterial co-infections and secondary infections are uncommon in patients with COVID-19 but may worsen outcomes. Assessment of bacterial complications is important in hospitalized patients with COVID-19, and the study findings are meaningful for the appropriate use of antimicrobial agents and management strategies.

**Supplementary Information:**

The online version contains supplementary material available at 10.1186/s12890-023-02418-3.

## Background

The coronavirus disease 2019 (COVID-19) caused by severe acute respiratory coronavirus 2 (SARS-CoV-2) has been spreading worldwide. It has been reported that some patients developed rapid respiratory failure approximately one week post disease onset [1] or thrombotic complications later [2]. Bacterial co-infections or secondary infections in patients with COVID-19 are considered uncommon complications [3] compared to the co-infection rate in patients with severe influenza which is reportedly 20–30% [4] and associated with higher mortality [5].

In a retrospective study of 989 COVID-19 patients, co-infections and secondary infections were found in 3.1 and 4.7% of patients, respectively [6]. The incidence of hospital-acquired secondary infection varies among studies, ranging from 13 to 27% in adults with SARS-CoV-2 [7].

In one meta-analysis, the most common bacterial pathogens associated with influenza are *Streptococcus pneumoniae* and *Staphylococcus aureus* in 35 and 28% of cases, respectively [8]. The most frequently identified bacterial co-infections in patients with COVID-19 are caused by *Staphylococcus aureus* and *Streptococcus pneumoniae* [6], whereas *Pseudomonas aeruginosa*, *Escherichia coli*, and *Staphylococcus aureus* are frequently isolated microorganisms in secondary infections [6, 9].

The characteristics of patients with COVID-19, that are also affected by bacterial infections, as well as the predictive factors for bacterial co-infections remain unclear. Thus, a more detailed analysis of COVID-19 cases with bacterial infections will contribute to not only clarifying the unknown aspects of SARS-CoV-2 infection but also promoting the appropriate treatment with antimicrobial agents.

The Japan COVID-19 Task Force is the largest Japanese cohort with biospecimen resources [10], and the findings from this database are considered reliable. In the present study, we evaluated the patient background, frequency, causative organisms, and clinical outcomes affected by bacterial infections in Japanese patients hospitalized for COVID-19.

## Methods

**Fig. 1:**
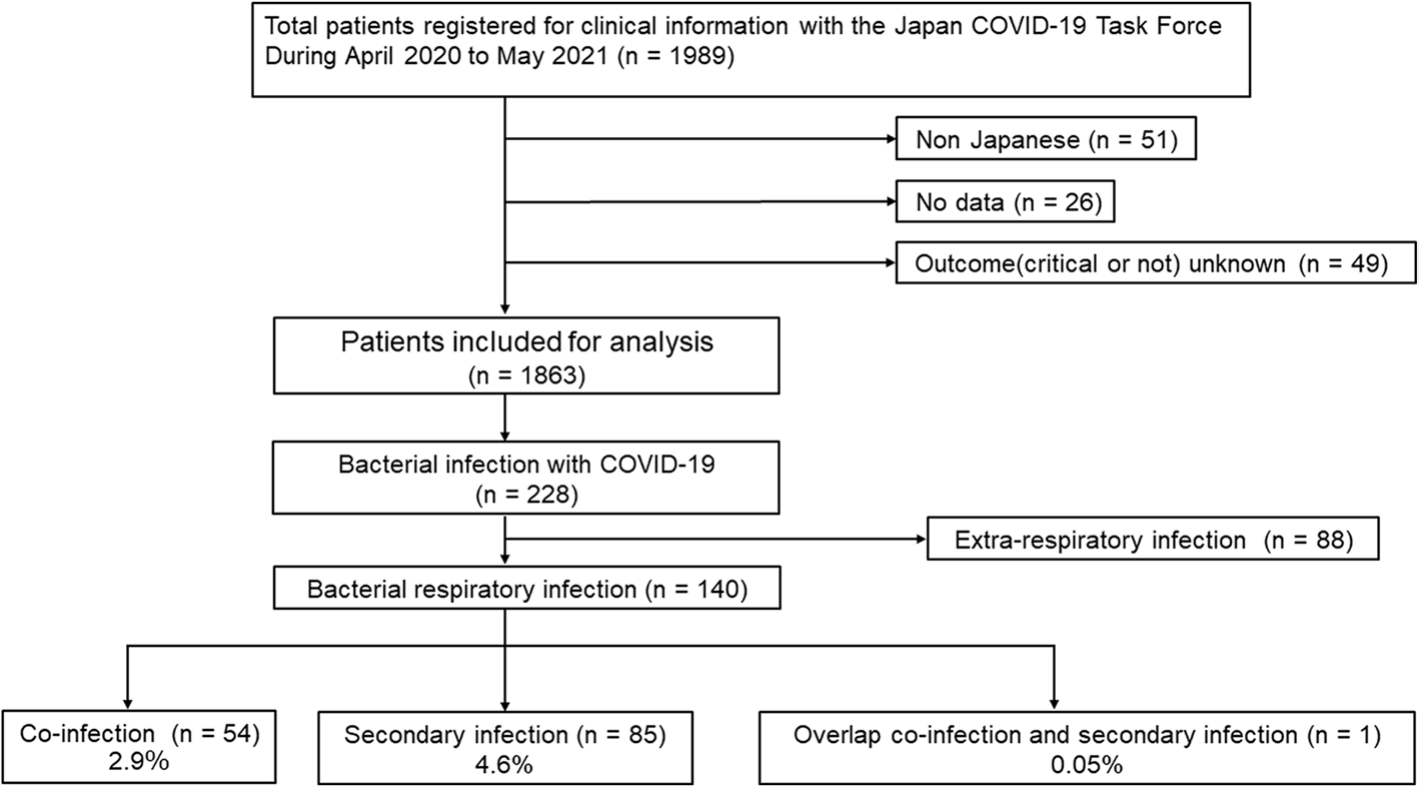
Study flow chart of patient identification and selection. Study flow chart of patient identification and selection. A total of 125 records were excluded from the 1,989 cases registered in the coronavirus disease 2019 (COVID-19) taskforce database for the following reasons: 51 were not Japanese, 26 lacked essential clinical information, and 49 had an unknown outcome. Ultimately, 1,863 cases met the eligibility criteria, of which 228 had complications from bacterial infections. Of these, 140 were respiratory bacterial infections, comprising 54 co-infections, 85 secondary infections, and one case of multiple infections

All patients were recruited through the Japan COVID-19 Task Force, and a retrospective cohort study was conducted [10, 11]. From April 2020 to May 2021, data from consenting consecutive inpatients aged 18 years or older who had been diagnosed with COVID-19 using SARS-CoV-2 polymerase chain reaction or antigen tests at one of the more than 100 affiliated hospitals were registered in an electronic case record form by the study subspecialist at the affiliated research institute. We excluded patients based on the following criteria: [i] non-Japanese patients and [ii] patients with incomplete medical records, such as inability to obtain critical outcome information (Fig. 1). We obtained written or oral informed consent from all enrolled patients, and the study was approved by the ethics committees of Keio University School of Medicine (20,200,061) and related research institutions.

### Data collection

The following information was extracted from the electronic case record form: age, sex, body mass index (BMI), clinical signs and symptoms, laboratory findings on admission, comorbidities, and disease severity (ICU entry, use of IMV, and survival status). All laboratory tests were performed according to the clinical care needs of the patients. Signs and symptoms were checked on admission, whereas other parameters, such as clinical course and treatment, were collected during hospitalization. Laboratory and radiographic results were collected within 48 h of the initial visit or admission. Missing data in the patient background were noted as unknown.

### Definitions

During hospitalization, standard microbiological tests were performed using sputum, urine, and blood samples. In patients receiving invasive mechanical ventilation, tracheal aspiration and bronchoalveolar lavage were performed as needed. The diagnosis of respiratory bacterial infection was determined by the attending clinician. Bacterial infections diagnosed at the time of admission due to COVID-19 or within 48 h of admission were defined as co-infections. Infections diagnosed after 48 h of COVID-19 admission were defined as secondary infections, as previously described [6].

### Outcome and statistics

The primary outcome was death. Continuous variables are presented as mean ± standard deviation, and categorical variables are presented as numbers (percentages). Data were compared among three groups defined by cases with co-infections, those with secondary infections, and cases without bacterial infections. ANOVA and Dunnet’s tests were used as indicated. To assess the impact of bacterial infection on COVID-19 severity, univariate and multivariate logistic regression analyses adjusted for patient characteristics, including BMI group, age, sex, chronic obstructive pulmonary disease (COPD), and presence of comorbidities associated with the severity of COVID-19 [12–18], were performed for ICU treatment, IMV use, and death. In intensive care, shock, IMV use, myocardial injury, acute kidney injury (AKI), and acute respiratory distress syndrome (ARDS) are also factors associated with death in COVID-19 [19–21]. In the multivariate analysis with death as the outcome, these variables were also added in a separate model. In the present analysis, shock was defined as cases in which patients were treated with vasopressors; for ARDS, these were substituted with cases with bilateral infiltrative shadows on chest radiographs. Odds ratio (OR) and adjusted OR are presented along with 95% confidence intervals. In all outcome analyses, the group without bacterial infection was defined as the control group. Moreover, univariate analysis was also performed for the frequency of thrombosis and myocardial injury during treatment, comparing the groups with and without bacterial infection as complications.

All P-values were two-sided, and statistical significance was set at *P* < 0.05. Data were analyzed using the JMP 16 program (SAS Institute Japan Ltd., Tokyo, Japan).

## Results

We assessed 1,989 consecutive adult patients with COVID-19 who had either been discharged or died during the study period at one of the hospitals that participated in the Japan COVID-19 Task Force. Of these, 1,863 patients with COVID-19 met the inclusion criteria, and 228 (12.2%) had bacterial infections concomitant with COVID-19. Respiratory bacterial infections with COVID-19 were diagnosed in 140 patients (7.5%), of which 54 (2.9%) were co-infections, and 85 (4.6%) were secondary infections. Only one case had an overlap of co-infection and secondary infection (Fig. 1).

### Patient characteristics at the time of admission

The main patient characteristics of each group are shown in Tables 1, 2, 3, and 4. Comparing the clinical backgrounds among the three groups comprising bacterial co-infections, secondary bacterial infections, and no bacterial infections, the parameters, such as age, COPD and percentage of steroid users were significantly higher in patients with bacterial infections than in those without (*P* < 0.05). The proportions of patients with male sex, hypertension, diabetes mellitus, chronic kidney disease, hyperuricemia, and cardiovascular disease, which are factors associated with the severity of COVID-19 [12–15, 18, 22], were exclusively increased in the group with secondary infections, as compared to that without bacterial infections (Table 1). Patients with bacterial co-infections showed significantly increased body temperatures at the time of admission (*P* < 0.05). In addition, patients with co-infections and secondary infections had higher prevalence of sputum (*P* = 0.005), disturbance of consciousness (*P* < 0.0001), and dyspnea (*P* < 0.0001). By contrast, the rates of dysgeusia and dysosmia were decreased in secondary infected patients, compared to the group without bacterial infections (*P* < 0.05) (Table 2).

**Table 1:** Patient characteristics at the time of admission

	No Bacterial Infection (*n* = 1635)	Respiratory Co-infection (*n* = 54)	Respiratory Secondary Infection (*n* = 85)	*P* value
Age	58.0 ± 18.0	65.9 ± 15.5	70.0 ± 13.9	< 0.0001^a=**/b=**^
Sex (male)	1131 (64.9%)	41 (75.9%)	71 (83.5%)	0.0006
BMI	24.5 ± 4.68	23.3 ± 4.57	24.6 ± 6.97	0.19
Smoker	260 (16.0%)	6 (13.6%)	5 (7.04%)	0.0001
Hypertension	607 (35.4%)	18 (34. 0%)	43 (51.2%)	0.02
Cardiovascular disease	167 (9.67%)	5 (9.26%)	23 (27.1%)	< 0.0001
Cancer	114 (6.65%)	5 (9.43%)	7 (8.43%)	0.61
Diabetes	381 (22.1%)	11 (20.4%)	32 (37.7%)	0.004
Asthma	112 (6.58%)	3 (5.88%)	5 (6.02%)	0.96
Hyperuricemia	178 (10.4%)	6 (11.1%)	20 (23.8%)	0.0001
COPD	74 (4.31%)	6 (11.3%)	8 (9.52%)	0.01
Chronic liver disease	53 (3.19%)	1 (1.89%)	4 (4.71%)	0.63
Chronic kidney disease	117 (7.09%)	6 (11.1%)	19 (22.6%)	< 0.0001
Steroid users	62 (3.60%)	7 (13.4%)	9 (10.6%)	< 0.0001
Immunosuppressive drug users	34 (1.98%)	0 (0%)	3 (3.53%)	0.35

Data are expressed as number (percentage) or mean ± standard deviation (SD)

**p < 0.01

^a^Comparison of patients without infection versus patients with community-acquired co-infection

^b^Comparison of patients without infection versus patients with hospital-acquired secondary infection

**Table 2:** Symptoms and vital signs at the time of admission

	No Bacterial Infection (*n* = 1635)	Respiratory Co-infection (*n* = 54)	Respiratory Secondary Infection (*n* = 85)	*P* value
Fever	37.2 ± 0.92	37.6 ± 1.03	37.4 ± 1.15	0.004 ^a=**^
Cough	1009 (58.8%)	27 (50.0%)	44 (58.7%)	0.43
Sputum	402 (23.5%)	21 (38.9%)	25 (33.8%)	0.005
Sore throat	409 (24.0%)	9 (17.0%)	9 (12.00%)	0.03
Nasal discharge	260 (15.2%)	4 (7.69%)	5 (6.67%)	0.04
Dysgeusia	318 (18.6%)	4 (7.84%)	6 (8.11%)	0.01
Dysosmia	283 (16.6%)	5 (9.80%)	4 (5.41%)	0.02
Dyspnea	513 (30.4%)	28 (51.9%)	35 (48.0%)	< 0.0001
Disturbance of consciousness	50 (2.91%)	8 (13.0%)	9 (12.2%)	< 0.0001
Malaise	835 (48.7%)	31 (57.4%)	42 (56.8%)	0.19
Systolic blood pressure	129.2 ± 19.6	129.8 ± 21.9	130.6 ± 27.4	0.83
Diastolic blood pressure	80.6 ± 13.5	79.6 ± 13.7	77.1 ± 13.9	0.09
Heart rate	87.2 ± 16.6	93.7 ± 19.6	86.7 ± 18.8	0.02^a=*^
Respiratory Rate	19.2 ± 4.58	20.7 ± 5.47	21.8 ± 6.47	< 0.0001^b=**^
SpO2	96.0 ± 3.07	93.7 ± 4.95	95.3 ± 3.04	< 0.0001^a=**^

Data are expressed as number (percentage) or mean ± SD

*p < 0.05

**p < 0.01

^a^Comparison of patients without infection versus patients with community-acquired co-infection

^b^Comparison of patients without infection versus patients with hospital-acquired secondary infection

**Table 3:** Serological data at the time of admission

	No Bacterial Infection (*n* = 1635)	Respiratory Co-infection (*n* = 54)	Respiratory Secondary Infection (*n* = 85)	*P* value
WBC (/μL)	5627.5 ± 2693.5	7579.8 ± 3868.5	7927.3 ± 4971.6	< 0.0001^a=**/b=**^
Neutrophil (/ μL)	3633.8 ± 2440.9	6109.9 ± 4275.7	6689.5 ± 4737.9	< 0.0001^a=**/b=**^
Lymphocyte(/μL)	1059.8 ± 616.3	705.2 ± 438.1	742.0 ± 462.9	< 0.0001^a=**/b=**^
Hb (g/dL)	14.1 ± 1.88	13.5 ± 1.98	13.1 ± 2.15	< 0.0001^a=*/b=**^
PLT (× 10^3^ /μL)	19.9 ± 7.65	20.4 ± 7.50	19.5 ± 8.95	0.80
Alb (g/dL)	3.78 ± 0.61	3.29 ± 0.92	3.12 ± 0.62	< 0.0001^a=**/b=**^
TB (mg/dL)	0.66 ± 0.36	0.78 ± 0.65	0.69 ± 0.43	0.08
ALP (IU/L)	175.5 ± 124.0	220.3 ± 142.3	197.3 ± 100.6	0.02^a=*^
AST (IU/L)	40.9 ± 69.1	54.7 ± 53.1	53.0 ± 91.3	0.13
ALT (IU/L)	38.6 ± 91.2	50.7 ± 63.5	43.8 ± 54.4	0.56
BUN (mg/dL)	16.5 ± 10.6	27.6 ± 25.5	31.4 ± 41.2	< 0.0001^a=**/b=**^
Cr (mg/dL)	1.06 ± 1.43	1.68 ± 2.34	1.38 ± 1.47	0.0018 ^a=**^
LDH (IU/L)	268.7 ± 136.6	337.1 ± 150.4	368.2 ± 184.1	< 0.0001^a=**/b=**^
UA (mg/dL)	4.88 ± 1.81	5.55 ± 2.34	5.11 ± 2.21	0.04^a=*^
CK (IU/L)	149.7 ± 456.3	248.7 ± 347.8	218.9 ± 387.1	0.14
Na (mEq/L)	138.3 ± 3.62	137.7 ± 5.53	137.4 ± 3.91	0.08
K (mEq/L)	3.98 ± 0.46	4.03 ± 0.54	4.20 ± 0.55	0.0002^b=**^
Cl (mEq/L)	101.8 ± 3.97	100.7 ± 5.56	102.1 ± 3.80	0.11
BNP (pg/mL)	69.4 ± 401.9	157.1 ± 403.0	104.2 ± 169.6	0.51
IgG(mg/dL)	1151.2 ± 359.4	1128.4 ± 293.3	1197.8 ± 293.6	0.27
Ferritin (ng/mL)	531.1 ± 583.0	814.4 ± 712.5	876.7 ± 982.0	< 0.0001^a=*/b=**^
KL-6 (IU/L)	315.9 ± 302.4	422.0 ± 308.9	488.2 ± 439.1	< 0.0001 ^b=**^
HbA1c (%)	6.37 ± 1.31	6.76 ± 1.60	6.88 ± 1.39	0.002 ^b=**^
CRP (mg/dL)	4.48 ± 5.35	10.8 ± 9.61	9.93 ± 10.1	< 0.0001^a=**/b=**^
Procalcitonin (ng/mL)	0.16 ± 0.62	2.81 ± 9.57	1.05 ± 2.83	< 0.0001^a=**/b=*^

Data are shown as mean ± SD

*WBC* white blood cell, *Hb* hemoglobin, *PLT* platelet, *Alb* albumin, *TB* total bilirubin, *ALP* alkaline phosphatase, *AST* aspartate aminotransferase, *ALT* alanine aminotransferase, *BUN* blood urea nitrogen, *Cr* creatinine, *LDH* lactate dehydrogenase, *UA* uric acid, *CK* creatinine kinase, *Na* sodium, *K* potassium, *Cl* chlorine, *BNP* brain natriuretic peptide, *IgG* Immunoglobulin G, *KL-6* Krebs von den Lungen-6, *CRP* C-reactive protein

*p < 0.05

**p < 0.01

^a^Comparison of patients without infection versus patients with community-acquired co-infection

^b^Comparison of patients without infection versus patients with hospital-acquired secondary infection

**Table 4:** Radiographic findings at the time of admission

	No Bacterial Infection (*n* = 1635)	Respiratory Co-infection (*n* = 54)	Respiratory Secondary Infection (*n* = 85)	*P* value
GGO on chest X-ray images	1176 (65.8%)	44 (83.0%)	70 (84.3%)	< 0.0001
Infiltrative shadow on chest X-ray images	423 (26.2%)	32 (64.0%)	45 (54.2%)	< 0.0001
GGO on chest CT images	1261 (81.2%)	47 (92.2%)	76 (97.4%)	0.0002
Infiltrative shadow on chest CT images	586 (38.4%)	31 (67.4%)	47 (61.8%)	< 0.0001

*GGO* ground glass opacity, *CT* Computed Tomography

### Patient laboratory and radiographic results at the time of admission

The clinical laboratory values of the enrolled patients are presented in Table 3. Patients with co-infections or secondary infections had higher levels of white blood cells, neutrophils, blood urea nitrogen, lactate dehydrogenase, serum ferritin, Krebs-von-den-Lungen-6, C-reactive protein, and procalcitonin (all *P* < 0.0001) than COVID-19 patients without bacterial infections. Conversely, lymphocytes, hemoglobin, and albumin values (all *P* < 0.0001) were significantly lower in patients with bacterial infections than in those without (Table 3). Regarding imaging findings on chest X-ray and chest CT scans, ground-glass opacities and infiltrated shadows were significantly more frequent in the groups with bacterial co-infections and secondary infections than in those without bacterial infections (all *P* < 0.0001) (Table 4).

### Causative pathogens of co-infections and secondary infections

Of the 55 co-infected patients with COVID-19, bacteria were detected in the sputum culture of 31 patients; bacterial species could not be isolated in the sputum culture of 24 patients, and for these patients, the diagnosis was based on clinical judgment.

**Fig. 2:**
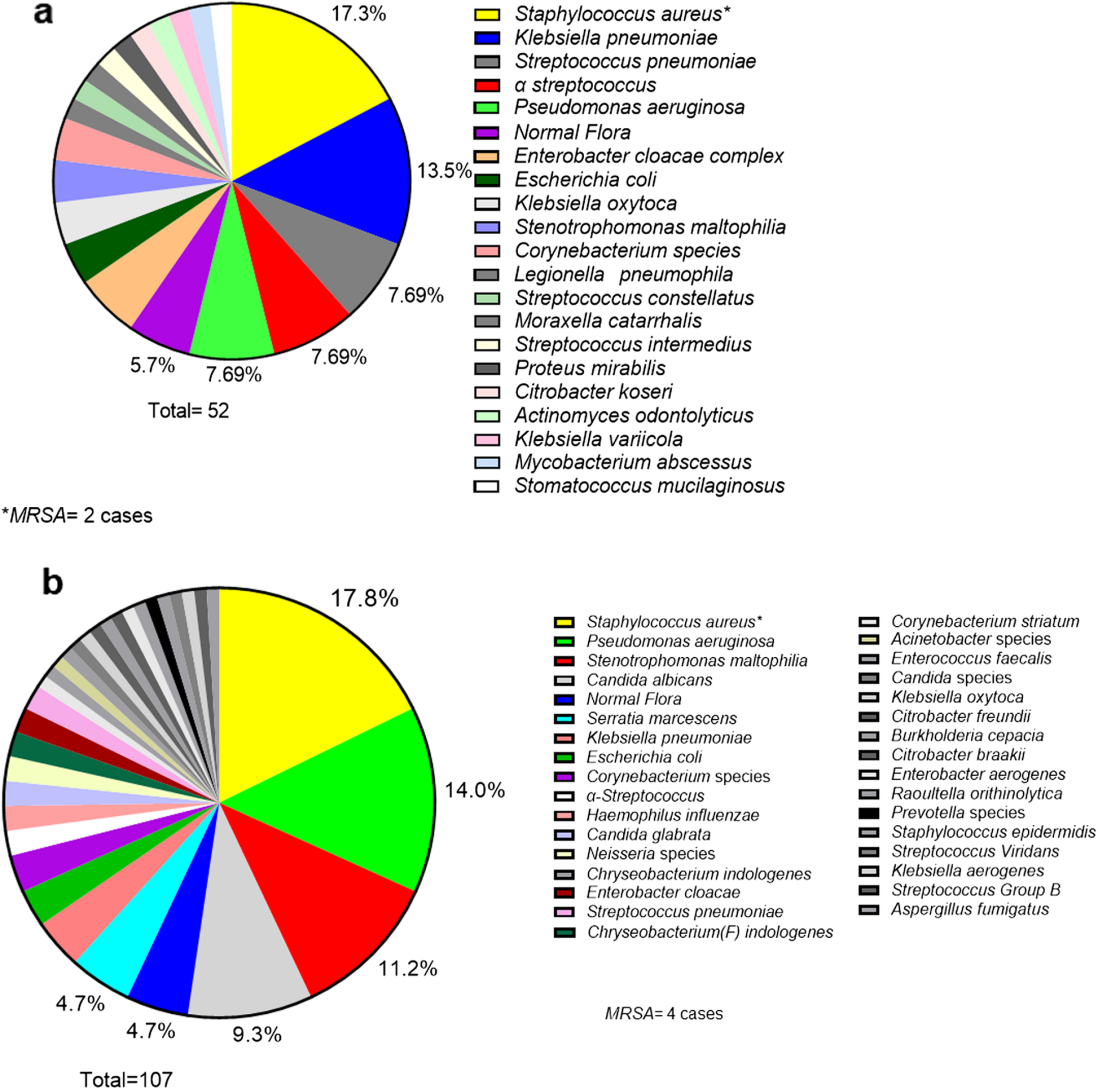
Bacterial pathogens identified in respiratory bacterial co-infection and secondary infection cases with coronavirus disease 2019. **a** All identified organisms as a proportion of total number of organisms per pathogen in sputum culture and urinary antigen of co-infection pneumonia with coronavirus disease 2019 (COVID-19). Bacterial pathogens detected in COVID-19 patients with respiratory bacterial co-infections, as a proportion (%) of the total number of isolates (*n* = 47). Some patients had multiple bacterial infection. In 24 cases, no causative organism was detected in sputum cultures. MRSA, *Methicillin-Resistant Staphylococcus aureus.* **b** All identified organisms as a proportion of total number of organisms per pathogen in sputum, tracheal aspirate, or bronchoalveolar lavage culture of secondary infection pneumonia with COVID-19. Bacterial pathogens detected in COVID-19 patients with respiratory bacterial secondary infection, as a proportion (%) of the total number of isolates (*n* = 107). Some patients had more than one bacterial infection. MRSA,* Methicillin-Resistant Staphylococcus aureus*

The most frequently isolated microorganisms in respiratory bacterial co-infections were *Staphylococcus aureus* (*n* = 9) and *Klebsiella pneumoniae* (*n* = 7) (Fig. 2a). Methicillin-resistant *Staphylococcus aureus* (MRSA) was isolated from two patients. One or more bacterial species were identified in the sputum culture of 82 of the 86 patients with secondary infection. No bacterial species were identified in the sputum culture of four patients. The most frequently isolated microorganisms were *Staphylococcus aureus* (*n* = 19), *Pseudomonas aeruginosa* (*n* = 15), and *Stenotrophomonas maltophilia* (*n* = 12). MRSA was detected in four cases. Figure 2b details the pathogens associated with respiratory bacterial secondary infections. Tracheal aspirates were obtained in most cases of ventilator-associated pneumonia (VAP); moreover, bronchoalveolar lavage was also performed to identify the causative organisms in one case. *Pseudomonas aeruginosa*, *Staphylococcus aureus*, and *Stenotrophomonas maltophilia* were frequently detected in these patients (Additional file 1).

### Predictive factors of respiratory co-infections

**Fig. 3:**
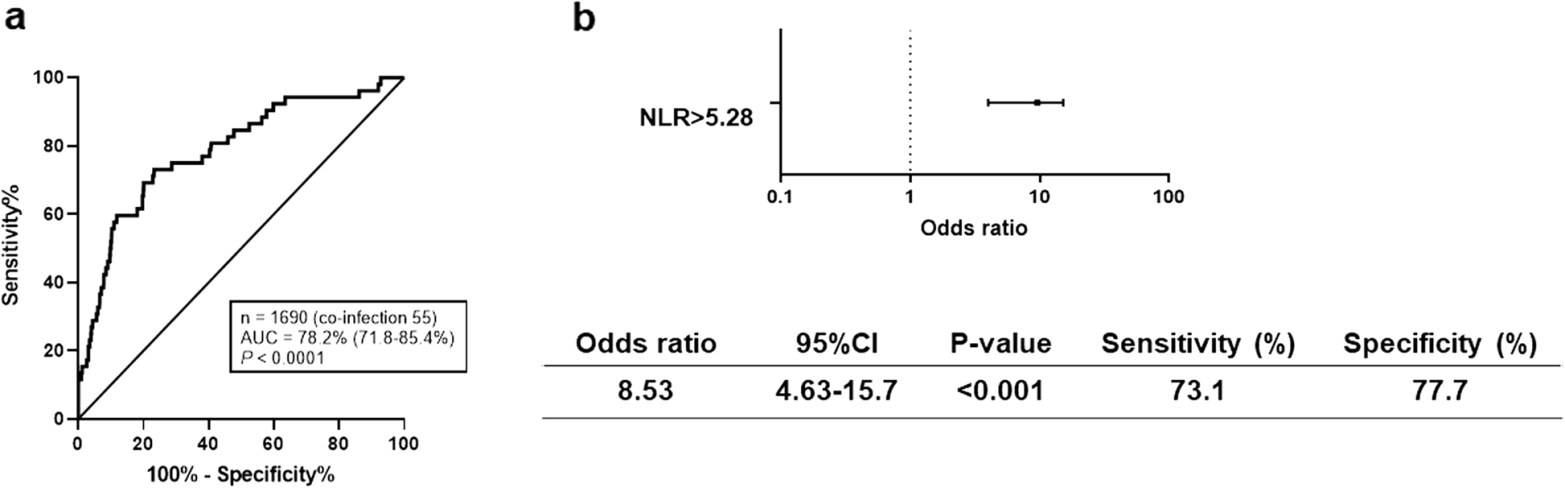
Predictive factor of respiratory co-infection. **a** Receiver operating characteristic curve for NLRs calculated from blood tests within 48 h of admission. **b** Sensitivity, specificity, and odds ratio for the NLR cutoff of 5.28 for complications of respiratory bacterial co-infections. CI, confidence interval; AUC, area under the curve

In laboratory parameters of co-infected patients, white blood cells, neutrophil count, lymphocyte count, neutrophil–lymphocyte ratio (NLR), C-reactive protein, and procalcitonin showed significant associations. The receiver operating characteristic curve was determined for each item, and the area under the curve (AUC) was calculated. The parameter NLR (AUC = 0.78) showed the highest value. The OR of NLR in patients with co-infections was 8.53 (4.63–15.7), with a sensitivity of 73.1% and a specificity of 77.7% at the cutoff value of 5.28 (Fig. 3, Additional file 2). Furthermore, we plotted ROC curves to determine predictors of co-infections using the NLR values in steroid users and non-users separately (Additional file 3). The results suggested NLR as a stronger predictive factor of co-infection complications in non-steroid users than in steroid users.

### Details of the respiratory secondary bacterial infections

Of the 86 secondary infection cases, 54 required the use of IMV, 41 of which developed VAP (Additional file 4a). Fourteen of the 41 VAP cases were repeated cases and had significantly higher mortality rates than those of non-repeated VAP and other secondary infection cases (Additional file 4b, c).

### Impact of bacterial infections on clinical prognosis

**Fig. 4:**
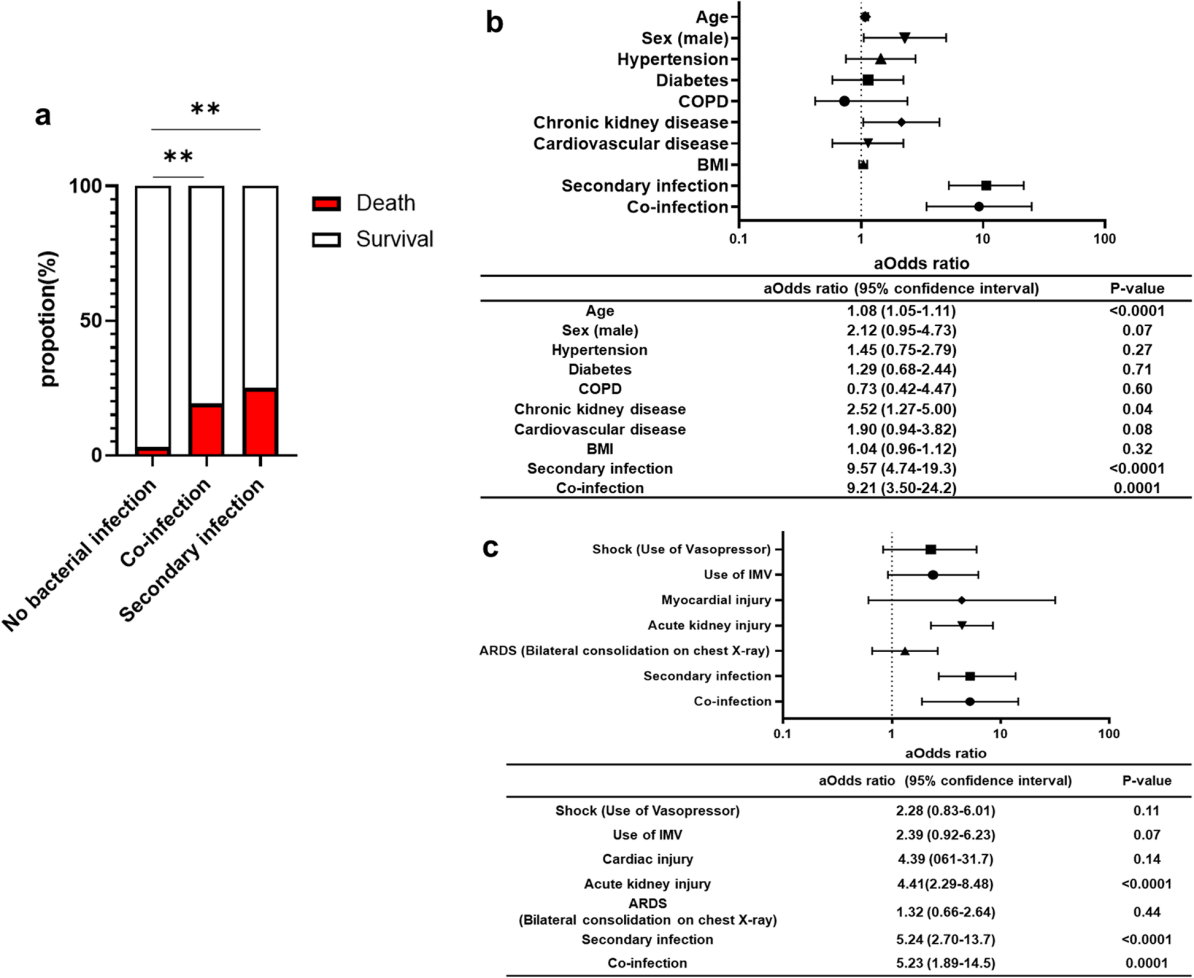
Clinical outcome of bacterial respiratory infection with COVID-19. **a** Proportions of deaths in bacterial co-infection, secondary infection, and non-bacterial infection cases with coronavirus disease 2019 (COVID-19) patients. **b** Forest plot of adjusted odds (aOdds) ratio via multivariate logistic regression analysis of risk factors of comorbidities associated with death in coronavirus disease 2019 (COVID-19) patients with bacterial infections. BMI, body mass index. **c** Forest plot of aOdds ratios via multivariate logistic regression analysis of risk factors of critical care category associated with death in coronavirus disease 2019 (COVID-19) patients with bacterial infections. IMV, invasive mechanical ventilation; ARDS, acute respiratory distress syndrome

Of the 55 co-infected patients, 18 (37.2%) were admitted to the ICU, 14 (25.5%) were intubated, and 10 (18.1%) died. Furthermore, among the 86 patients with secondary infections, 58 (67.4%) were admitted to the ICU, 54 (62.8%) were intubated, and 63 (75.0%) died. In the univariate analysis, patients with co-infections or secondary infections had higher mortality rates than those without bacterial infections (Fig. 4a). Multivariate logistic regression analysis showed that compared to patients without bacterial infections, those who had co-infections (OR = 9.21 (3.50–24.2)) or secondary infections (OR = 9.57 (4.74–19.3)) were at higher risk of death (Fig. 4b). Moreover, both co-infections (OR = 5.23 (1.89–14.5)) and secondary infections (OR = 5.24 (2.70–13.7)) were risk factors associated with death in the multivariate analysis of model 2, which included IMV use and the complications of AKI, myocardial injury, shock, and ARDS during hospitalization as variables (Fig. 4c). In this study, 141 patients were treated with anti-IL6 receptor antibodies as immunosuppressive therapy. The incidence of secondary infections was significantly increased in the group treated with anti-IL6 receptor antibodies (Additional file 5a). However, in the multivariate analysis, the use of anti-IL6 receptor antibodies was not associated with death (Additional file 5b).

The univariate analysis also showed that patients with co-infections or secondary infections excluding VAP were more frequently admitted to the ICU and underwent IMV more frequently compared to those without bacterial infections. Both univariate analyses of ICU admission and IMV use were performed in cases of secondary infection occurring prior to these events (Additional file 6a, b). In a multivariate analysis that evaluated risk factors for ICU admission and IMV use, secondary infection excluding VAP and co-infection were independent risk factors for these severe conditions (Additional file 6c). Furthermore, the multivariate analysis of risk factors for death with nosocomial pneumonia and VAP separately revealed that both were independent risk factors (Additional file 7).

The incidence of thrombosis and myocardial injury significantly increased in both the co-infection and secondary infection groups compared to those in the non-bacterial infection group (Additional file 8).

## Discussion

In this study, we present a large series of Japanese patients with COVID-19 focusing on the description of respiratory bacterial community-acquired co-infections and hospital-acquired secondary infections in these patients. The incidence of complications from bacterial pneumonia in patients hospitalized due to COVID-19 was less than that from influenza [23]. The frequency of SARS-CoV-2 co-infections with other respiratory pathogens has been reported to be generally low [1, 23]; in the present study of Japanese patients, the rate of respiratory bacterial co-infections was 3.0%. The incidence of bacterial infections secondary to COVID-19 varies in previous studies [6, 23, 24]. In this study, the frequency of secondary infection was 4.6%. The frequency of both co-infections and secondary infections in a previous Japanese study is comparable to that in this study (2.86 and 5.59% vs. 3.0 and 4.6%, respectively) [24].

Interestingly, severity-associated comorbidities such as hypertension, diabetes, and chronic kidney disease were frequently observed in secondary infection cases. For patients with these predisposing factors, it is important to consider the possibility complications associated with secondary bacterial infections. We have shown, using multivariate analysis, that both co-infections and secondary infections were associated with death at a high odds ratio independent of other severity factors. These facts indicate that it is crucial for clinicians to properly assess bacterial complications and initiate appropriate antibiotic therapy.

Among cases with respiratory bacterial co-infections in this study, *Staphylococcus aureus*, *Klebsiella pneumoniae*, and other causative organisms like those previously reported were detected in sputum cultures [6, 25]. *Staphylococcus aureus* is a frequent concomitant in patients with influenza infection [8]. Previous studies have hypothesized that excessive use of broad-spectrum antibiotics, especially for early bacterial co-infections, can induce changes in the microbiota, leading to VAP in the late stages of hospitalization with poor prognosis [9]. Therefore, the choice of antibiotics in the early stage of the treatment is important. If bacterial co-infections are suspected, the use of antibiotics with coverage of *Staphylococcus aureus* is recommended. There is an urgent need to develop prospective evidence to support the development of antimicrobial strategies and appropriate stewardship interventions specific to COVID-19 treatment, as previously suggested [26].

The frequency of co-infections and secondary infections associated with COVID-19, the identification of their causative organisms, and their association with clinical outcomes [7, 27]. However, in addition to these things, we consider this study valuable as we simultaneously revealed the clinical characteristics of co-infection and secondary infection cases and identified the utility of NLR as a diagnostic parameter for co-infection.

At the time of diagnosis, it is often difficult to discriminate between SARS-CoV-2 infection alone and SARS-CoV-2 infection with concomitant bacterial pneumonia. It remains controversial whether antimicrobial agents should be routinely used. In our study, the parameter NLR was found to be the best indicator of complications from bacterial co-infection with COVID-19 (cutoff, 5.28; AUC, 0.78; sensitivity, 73.1%; specificity, 77.7%). An increase in NLR can be caused by a systemic inflammatory response that disrupts the immunological functions of the body, resulting in a simultaneous increase in neutrophil count and decrease in lymphocyte count. NLR is an indicator of systemic inflammation and is widely used in many diseases, including the prediction of mortality in septic patients [28]. A meta-analysis of multiple observational studies showed that a high NLR is associated with severity and high mortality in COVID-19 [29], possibly attributed to mechanisms by which inflammatory factors, such as interleukin-6, interleukin-8, and granulocyte colony-stimulating factor, that are elicited in SARS-CoV-2 infections may stimulate the production of neutrophils [30].

The increased NLR in COVID-19 patients with bacterial infections might also be the result of a more aggressive neutrophil migration induced by immune responses to bacteria. Various previous studies have suggested optimal cutoff values for the NLR. Four studies aiming at the prediction of illness severity determined differing values ranging from 3.3 to 5.9 [30–32]. Two additional studies that sought to predict mortality established NLR cutoff values of 7.9 and 11.8 [33, 34]. In the present study, the best cutoff value of NLR for predicting complications of respiratory bacterial co-infections was 5.28, which is slightly higher than the value for predicting COVID-19 severity and lower than the value for death in patients with COVID-19. We found that respiratory bacterial co-infections in patients with COVID-19 were associated with ICU admission, IMV use, and high mortality. Thus, the cutoff value of 5.28 for NLR in this study is reasonable. Compared to other prognostic parameters for COVID-19, such as interleukin-6, D-dimers, C-reactive protein, and erythrocyte sedimentation rate [35, 36], NLR is more practical for clinical application since it can easily be obtained via routine blood examinations.

Our study is the first to suggest that NLR may help diagnose cases of bacterial co-infection in patients with COVID-19, and our calculated NLR cutoff value will be useful in making decisions regarding the concomitant use of antibiotics. Importantly, since steroids increase blood neutrophil counts and decrease lymphocyte counts, an elevated NLR can be confused with an infectious complication. In this study, the predictive accuracy of co-infection by NLR in the non-steroid users was superior to that in steroid users, suggesting that clinicians need to be cautious in this regard.

Regarding the organisms isolated in cases with secondary infections, the same previously reported species, such as *Staphylococcus aureus*, *Pseudomonas aeruginosa*, and *Stenotrophomonas maltophilia,* were detected [6]. Infections with *Staphylococcus aureus* secondary to influenza infections are common, which may be due to excessive mucus secretion, decreased clearance of mucosal cilia, and destruction of epithelial cells [8]. The mechanisms involved in secondary *Staphylococcus aureus* infections following SARS-CoV-2 infection remain poorly understood and require further research.

In a study analyzing the association between upper respiratory tract flora and clinical prognosis in patients with COVID-19, *streptococcus*-dominated microbiota was associated with lower severity of illness and lower mortality [37]. However, how the microbiota of the respiratory tract is altered by COVID-19 remains largely unknown [38]. Many of the organisms isolated from the sputum of secondary infection cases in our study are known to cause hospital-acquired pneumonia. Since secondary infections are independently associated with poor outcomes such as high mortality, further research, especially on how bacterial infections occur during hospitalization, is essential to reduce the incidence of secondary infections and provide appropriate therapeutic management.

Our study has several limitations. First, this study included only hospitalized patients with COVID-19, which might have resulted in a biased sample due to the high severity of the disease. Second, the diagnosis of bacterial complications was entirely dependent on the clinical judgment of the physician, and microbiological tests were not necessarily required to prove the causative pathogens. Two studies that strictly analyzed the frequency of VAP and causative organisms in patients who required IMV reported much higher rates of bacterial co-infections compared to those reported by studies that employed only the usual methods of care to identify bacterial pathogens [39]. This is a major limitation of our study. Regrettably, we were unable to prospectively assess all cases for the rigorous approach to detect bacterial pathogens in this multicenter study. Moreover, bacteriological evaluation, including evaluation of urinary antigens and endotracheal aspiration, as well as molecular biological evaluation, such as comprehensive viral and bacterial PCR tests, were not performed; thus, the identification of the causative organisms might have been underestimated. Third, there might not have been standardized management practices among different facilities during the study period. In particular, antibiotic administration and treatment methods have not been standardized, which may have affected the clinical outcomes. Fourth, this study included only Japanese patients; therefore, it may be inappropriate to generalize or extrapolate the present results to populations of patients with COVID-19 in other countries. Fifth, the sample size of this study was determined incidentally and was not designed to be statistically sufficient to detect the effects of complications from bacterial infections on severe illness and death. Further studies are needed to address these limitations and develop optimal treatment strategies.

## Conclusions

In this study, we determined the frequency of bacterial co-infections and secondary infections in Japanese patients with COVID-19 and the causative organisms, as well as their associations with clinical outcomes. We also revealed the characteristics of patients with bacterial co-infections and secondary infections. Although co-infections and secondary infections in COVID-19 are infrequent, they are significantly associated with disease severity and high mortality, suggesting that the evaluation of bacterial infections and appropriate antimicrobial therapies are crucial in the treatment of COVID-19. Notably, our results suggest that NLR may be useful in the diagnosis of bacterial co-infections with COVID-19.

## Supplementary Information

**Additional file 1.** Identification of organisms in ventilator-associated pneumoniacases

**Additional file 2.** Evaluation of white blood cells, neutrophils, lymphocytes, neutrophil-lymphocyte ratio, C-reactive protein, and procalcitonin on admission as predictors of respiratory bacterial co-infection based on the area under the curve

**Additional file 3.** Neutrophil-lymphocyte ratioas a predictor of co-infection in steroid and non-steroid users

**Additional file 4.** Details of respiratory secondary infection.

**Additional file 5.** Association of anti-IL-6 receptor antibody use with incidence of secondary infection and death. 

**Additional file 6.** Admission to intensive care unitand use of invasive mechanical ventilationof bacterial respiratory infection with coronavirus disease 2019.

**Additional file 7.** Forest plot of adjusted oddsratios by multivariate logistic regression analysis of risk factors of death in patients of coronavirus disease 2019with secondary infection except ventilator-associated pneumoniaand VAP.

**Additional file 8.** Proportion of thrombosis and myocardial injury in bacterial respiratory co-infection and secondary infection with coronavirus disease 2019.

## Data Availability

The data that support the findings of this study are available from the corresponding author upon reasonable request.

## Abbreviations

COVID-19: Coronavirus disease 2019
SARS-CoV-2: Severe acute respiratory coronavirus 2
ICU: Intensive care unit
IMV: Invasive mechanical ventilation
ARDS: Acute respiratory distress syndrome
BMI: Body mass index
OR: Odds ratio
COPD: Chronic obstructive pulmonary disease
MRSA: Methicillin-resistant *Staphylococcus aureus*
VAP: Ventilator-associated pneumonia
NLR: Neutrophil-lymphocyte ratio
AUC: Area under the curve
