# Supplementary material for: Impact of respiratory bacterial infections on mortality in Japanese patients with COVID-19: a retrospective cohort study
Source: BMC Pulm Med. 2023 Apr 26;23:146. doi: 10.1186/s12890-023-02418-3 (PMC10131342; doi:10.1186/s12890-023-02418-3)
Supplement: Supplementary file 1 — Additional file 1. Identification of organisms in ventilator-associated pneumoniacases [file 12890_2023_2418_MOESM1_ESM.docx]

# Additional Files

**Impact of respiratory bacterial infections on mortality in Japanese patients with COVID-19: a retrospective cohort study**

Kensuke Nakagawara^1^, Hirofumi Kamata^1^*, Shotaro Chubachi^1^, Ho Namkoong^2^, Hiromu Tanaka^1^, Ho Lee^1^, Shiro Otake^1^, Takahiro Fukushima^1^, Tatsuya Kusumoto^1^, Atsuho Morita^1^, Shuhei Azekawa^1^, Mayuko Watase^1^, Takanori Asakura^1,3,4^, Katsunori Masaki^1^, Makoto Ishii^1,5^, Akifumi Endo^6^, Ryuji Koike^6^, Hiroyasu Ishikura^7^, Tohru Takata^8^, Yasushi Matsushita^9^, Norihiro Harada^10^, Hiroyuki Kokutou^11^, Takashi Yoshiyama^11^, Kensuke Kataoka^12^, Yoshikazu Mutoh^13^, Masayoshi Miyawaki^14^, Soichiro Ueda^14^, Hiroshi Ono^15^, Takuya Ono^16^, Tomohisa Shoko^16^, Hiroyuki Muranaka^17^, Kodai Kawamura^17^, Nobuaki Mori^18^, Takao Mochimaru^19^, Mototaka Fukui^20^, Yusuke Chihara^20^, Yoji Nagasaki^21^, Masaki Okamoto^21^, Masaru Amishima^22^, Toshio Odani^23^, Mayuko Tani^24^, Koichi Nishi^24^, Yuya Shirai^25^, Ryuya Edahiro^25^, Akira Ando^26^, Naozumi Hashimoto^26^, Shinji Ogura^27^, Yuichiro Kitagawa^27^, Toshiyuki Kita^27^, Takashi Kagaya^28^, Yasuhiro Kimura^29^, Naoki Miyazawa^29^, Tomoya Tsuchida^30^, Shigeki Fujitani^31^, Koji Murakami^32^, Hirohito Sano^32^, Yuki Sato^33^, Yoshinori Tanino^33^, Ryo Otsuki^34^, Shuko Mashimo^34^, Mizuki Kuramochi^35^, Yasuo Hosoda^35^, Yoshinori Hasegawa^36^, Tetsuya Ueda^36^, Yotaro Takaku^37^, Takashi Ishiguro^37^, Akiko Fujiwara^38^, Naota Kuwahara^38^, Hideya Kitamura^39^, Eri Hagiwara^39^, Yasushi Nakamori^40^, Fukuki Saito^40^, Yuta Kono^41^, Shinji Abe^41^, Tomoo Ishii^42^, Takehiko Ohba^43^, Yu Kusaka^43^, Hiroko Watanabe^44^, Makoto Masuda^45^, Hiroki Watanabe^45^, Yoshifumi Kimizuka^46^, Akihiko Kawana^46^, Yu Kasamatsu^47^, Satoru Hashimoto^48^, Yukinori Okada^49-51^, Tomomi Takano^52^, Kazuhiko Katayama^53^, Masumi Ai^54^, Atsushi Kumanogoh^25^, Toshiro Sato^55^, Katsushi Tokunaga^56^, Seiya Imoto^57^, Yuko Kitagawa^58^, Akinori Kimura^59^, Satoru Miyano^60^, Naoki Hasegawa^2^, Seishi Ogawa^61^, Takanori Kanai^62^, Koichi Fukunaga^1^, and The Japan COVID-19 Task Force.

**
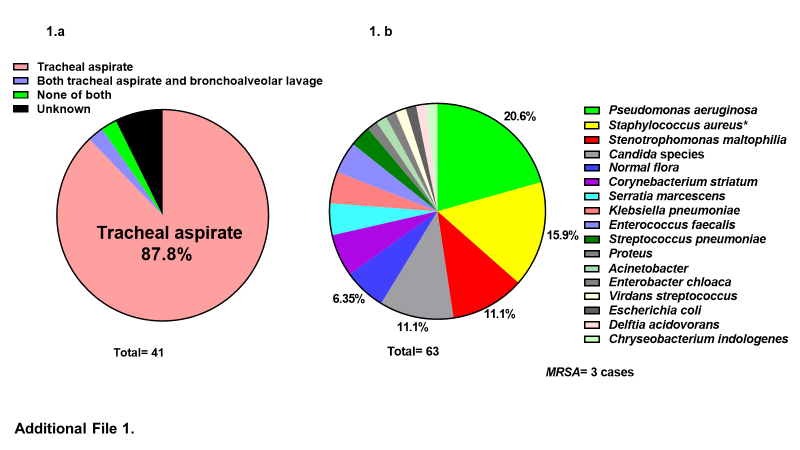
**

**Additional File 1. Identification of organisms in ventilator-associated pneumonia (VAP) cases. a** Method for identification of causative organisms in cases of VAP

Tracheal aspirates obtained in 37 of 41 cases of VAP, of which in 1 case, bronchoalveolar lavage was also performed along with tracheal aspirate. **b** Identified organisms as a proportion of the total number of organisms per pathogen in sputum culture by bronchoalveolar lavage and tracheal aspirate of ventilator-associated pneumonia with coronavirus disease 2019 (COVID-19).

Bacterial pathogens detected in COVID-19 patients with VAP, as a proportion (%) of the total number of isolates (n = 63). Some patients had multiple bacterial infection.
