# Supplementary material for: Impact of respiratory bacterial infections on mortality in Japanese patients with COVID-19: a retrospective cohort study
Source: BMC Pulm Med. 2023 Apr 26;23:146. doi: 10.1186/s12890-023-02418-3 (PMC10131342; doi:10.1186/s12890-023-02418-3)
Supplement: Supplementary file 2 — Additional file 2. Evaluation of white blood cells, neutrophils, lymphocytes, neutrophil-lymphocyte ratio, C-reactive protein, and procalcitonin on admission as predictors of respiratory bacterial co-infection based on the area under the curve [file 12890_2023_2418_MOESM2_ESM.docx]

**Additional File 2.** Evaluation of white blood cells (WBC), neutrophils, lymphocytes, neutrophil-lymphocyte ratio (NLR), C-reactive protein (CRP), and procalcitonin on admission as predictors of respiratory bacterial co-infection based on the area under the curve (AUC)

|  | AUC | 95% confidence interval | P value |
| --- | --- | --- | --- |
| WBC (/μL) | 0.70 | 0.63-0.78 | < 0.0001 |
| Neutrophil (/μL) | 0.69 | 0.62-0.76 | < 0.0001 |
| Lymphocyte (/μL) | 0.70 | 0.63-0.76 | < 0.0001 |
| NLR | 0.78 | 0.72-0.85 | < 0.0001 |
| CRP (mg/dL) | 0.75 | 0.69-0.80 | < 0.0001 |
| Procalcitonin (ng/mL) | 0.72 | 0.65-0.79 | < 0.0001 |
