# Supplementary material for: Impact of respiratory bacterial infections on mortality in Japanese patients with COVID-19: a retrospective cohort study
Source: BMC Pulm Med. 2023 Apr 26;23:146. doi: 10.1186/s12890-023-02418-3 (PMC10131342; doi:10.1186/s12890-023-02418-3)
Supplement: Supplementary file 3 — Additional file 3. Neutrophil-lymphocyte ratioas a predictor of co-infection in steroid and non-steroid users [file 12890_2023_2418_MOESM3_ESM.docx]

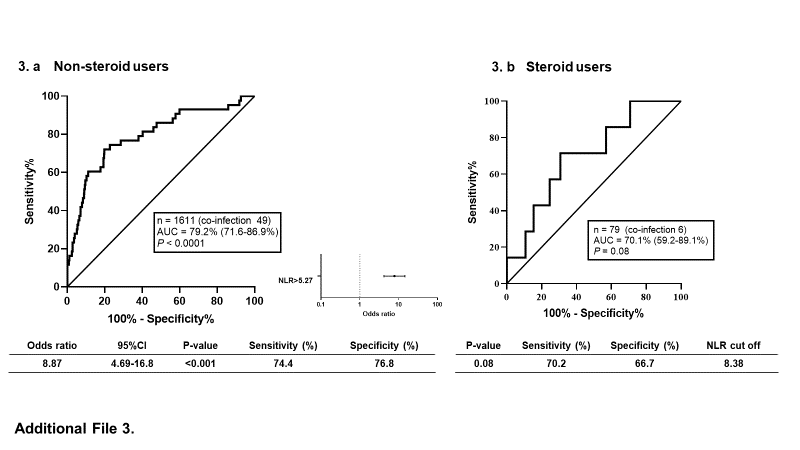


**Additional File 3. Neutrophil-lymphocyte ratio (NLR) as a predictor of co-infection in steroid and non-steroid users. a, b** Receiver operating characteristic curve for NLR calculated from blood tests within 48 h of admission in steroid and non-steroid users. Sensitivity, specificity, and odds ratio for the NLR cutoff of 5.27 for complications of respiratory bacterial co-infections with and without steroid users are shown. CI, confidence interval; AUC, area under the curve.
