# Supplementary material for: Impact of respiratory bacterial infections on mortality in Japanese patients with COVID-19: a retrospective cohort study
Source: BMC Pulm Med. 2023 Apr 26;23:146. doi: 10.1186/s12890-023-02418-3 (PMC10131342; doi:10.1186/s12890-023-02418-3)
Supplement: Supplementary file 4 — Additional file 4. Details of respiratory secondary infection. [file 12890_2023_2418_MOESM4_ESM.docx]

**
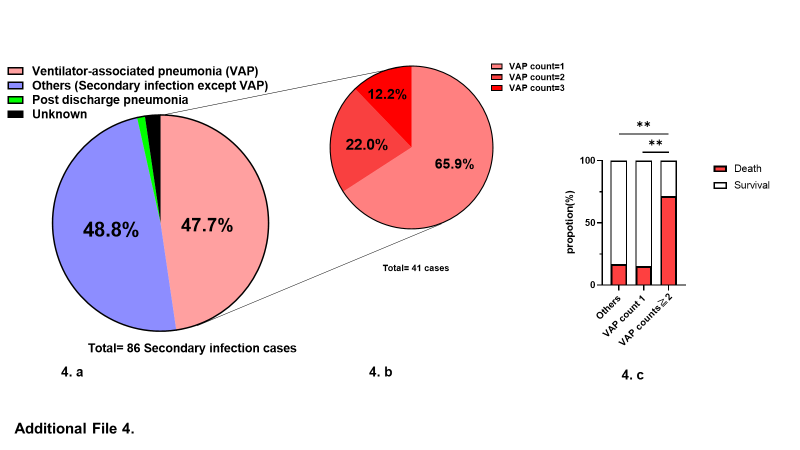
**

**Additional File 4. Details of respiratory secondary infection. a** Proportion of ventilator-associated pneumonia and other secondary pneumonia in respiratory secondary infection cases. **b** Number of repeat cases of ventilator-associated pneumonia. VAP, ventilator-associated pneumonia. **c** Comparison of mortality between number of repeat ventilator-associated pneumonia and other hospital-acquired pneumonia.
