# Supplementary material for: Impact of respiratory bacterial infections on mortality in Japanese patients with COVID-19: a retrospective cohort study
Source: BMC Pulm Med. 2023 Apr 26;23:146. doi: 10.1186/s12890-023-02418-3 (PMC10131342; doi:10.1186/s12890-023-02418-3)
Supplement: Supplementary file 5 — Additional file 5. Association of anti-IL-6 receptor antibody use with incidence of secondary infection and death. [file 12890_2023_2418_MOESM5_ESM.docx]

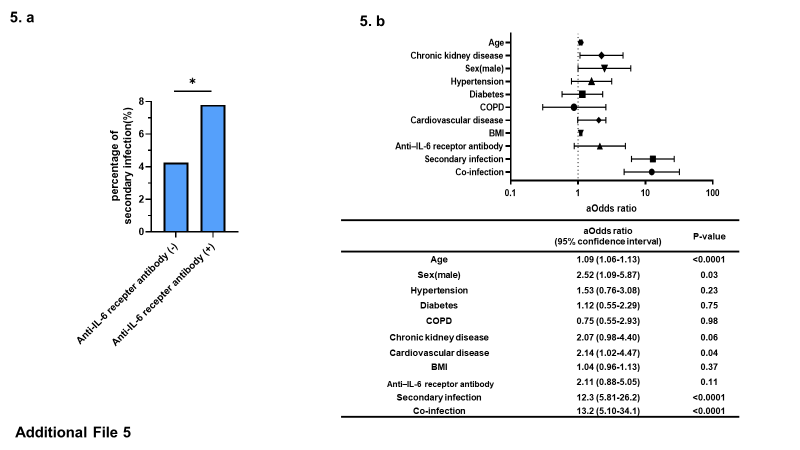


**Additional File 5. Association of anti-IL-6 receptor antibody use with incidence of secondary infection and death. a** Results of the univariate analysis for respiratory secondary infection. Percentage of respiratory secondary infection in patients treated with and without anti-IL-6 receptor antibody. *p < 0.05**.** **b** Multiple logistic analysis of risk factors of death. Forest plots of adjusted odds ratios (aORs) obtained from multivariate logistic regression analysis of risk factors of death in COVID-19 patients with bacterial infections. In addition to co-infection and secondary infection, age, sex, BMI, diabetes, hypertension, COPD, chronic kidney disease, cardiovascular disease, and anti-IL-6 receptor antibody were included as variables in the multivariate analysis. BMI, body mass index.
