# Supplementary material for: Impact of respiratory bacterial infections on mortality in Japanese patients with COVID-19: a retrospective cohort study
Source: BMC Pulm Med. 2023 Apr 26;23:146. doi: 10.1186/s12890-023-02418-3 (PMC10131342; doi:10.1186/s12890-023-02418-3)
Supplement: Supplementary file 6 — Additional file 6. Admission to intensive care unitand use of invasive mechanical ventilationof bacterial respiratory infection with coronavirus disease 2019. [file 12890_2023_2418_MOESM6_ESM.docx]

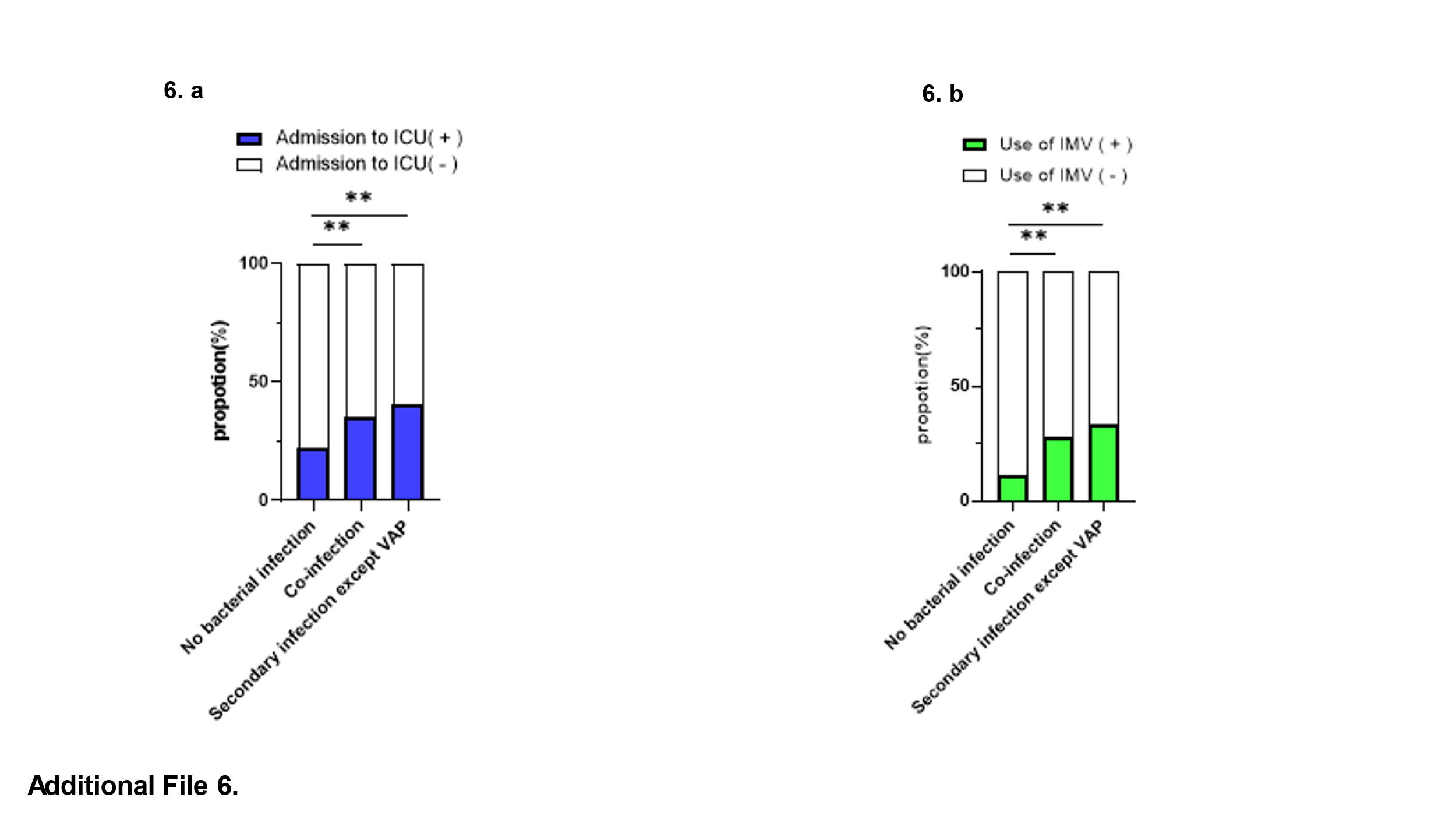


**
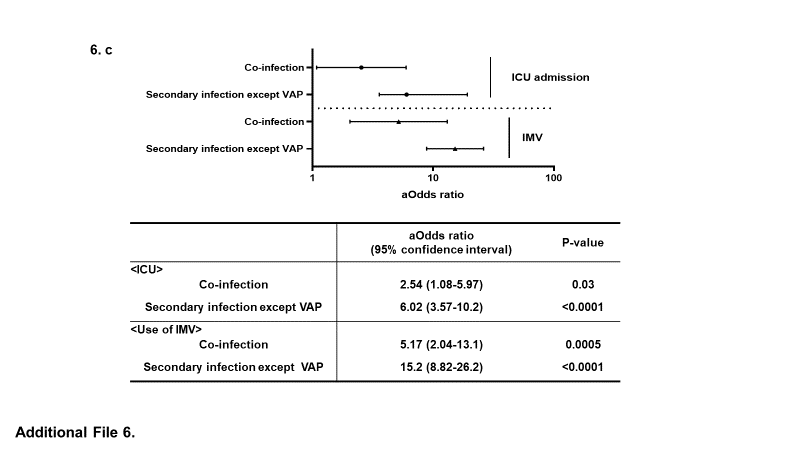
**

**Additional File 6.** **Admission to intensive care unit (ICU) and use of invasive mechanical ventilation (IMV) of bacterial respiratory infection with coronavirus disease 2019 (COVID-19). a, b** Results of the univariate analysis for ICU admission and use of IMV. Proportions of ICU admissions (a) and use of IMV (b) in bacterial co-infection, secondary infection except ventilator-associated pneumonia (VAP), and non-bacterial infection cases with coronavirus disease 2019 (COVID-19). **p < 0.01**. c** Multiple logistic analysis of risk factors of ICU admission and use of IMV. Forest plots of adjusted odds (aOdds) ratios using multivariate logistic regression analysis of risk factors of ICU admission and use of IMV in COVID-19 patients with bacterial infections. In addition to co-infection and secondary infection, age, sex, BMI, diabetes, hypertension, COPD, chronic kidney disease, and cardiovascular disease were used as variables in the multivariate analysis. BMI, body mass index.
