# Supplementary material for: Impact of respiratory bacterial infections on mortality in Japanese patients with COVID-19: a retrospective cohort study
Source: BMC Pulm Med. 2023 Apr 26;23:146. doi: 10.1186/s12890-023-02418-3 (PMC10131342; doi:10.1186/s12890-023-02418-3)
Supplement: Supplementary file 7 — Additional file 7. Forest plot of adjusted oddsratios by multivariate logistic regression analysis of risk factors of death in patients of coronavirus disease 2019with secondary infection except ventilator-associated pneumoniaand VAP. [file 12890_2023_2418_MOESM7_ESM.docx]

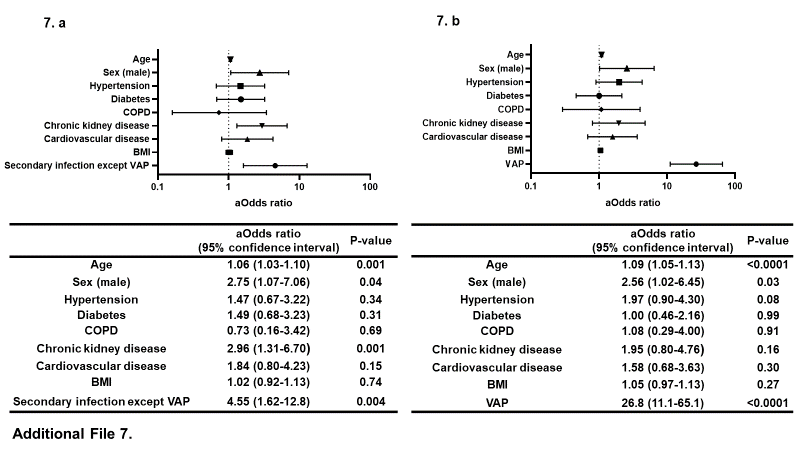


**
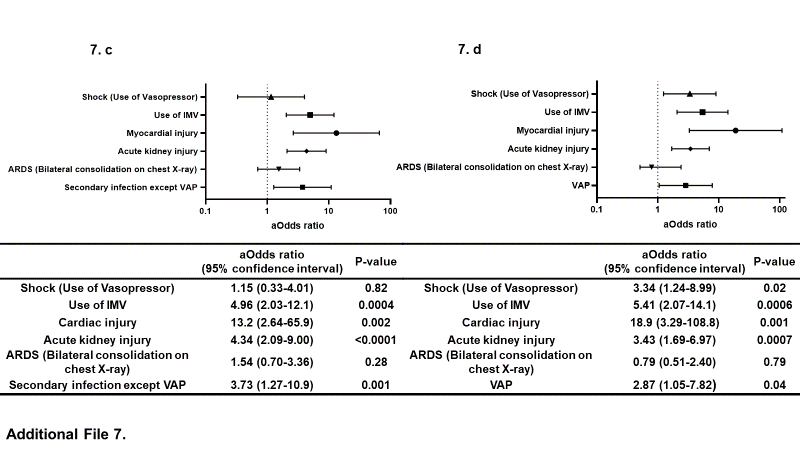
 Additional File 7**. **Forest plot of adjusted odds (aOdds) ratios using multivariate logistic regression analysis of risk factors of death in coronavirus disease 2019 (COVID-19) patients with secondary infection except ventilator-associated pneumonia (VAP) and VAP. a, b** Forest plot of adjusted odds (aOdds) ratios using multivariate logistic regression analysis of risk factors of comorbidities associated with death in COVID-19 patients with secondary infection except VAP and VAP. BMI, body mass index. **c, d** Forest plot of aOdds ratios using multivariate logistic regression analysis of risk factors of critical care category associated with death in COVID-19 patients with secondary infection except VAP and VAP. IMV, invasive mechanical ventilation; ARDS, acute respiratory distress syndrome.
