# Supplementary material for: Impact of respiratory bacterial infections on mortality in Japanese patients with COVID-19: a retrospective cohort study
Source: BMC Pulm Med. 2023 Apr 26;23:146. doi: 10.1186/s12890-023-02418-3 (PMC10131342; doi:10.1186/s12890-023-02418-3)
Supplement: Supplementary file 8 — Additional file 8. Proportion of thrombosis and myocardial injury in bacterial respiratory co-infection and secondary infection with coronavirus disease 2019. [file 12890_2023_2418_MOESM8_ESM.docx]

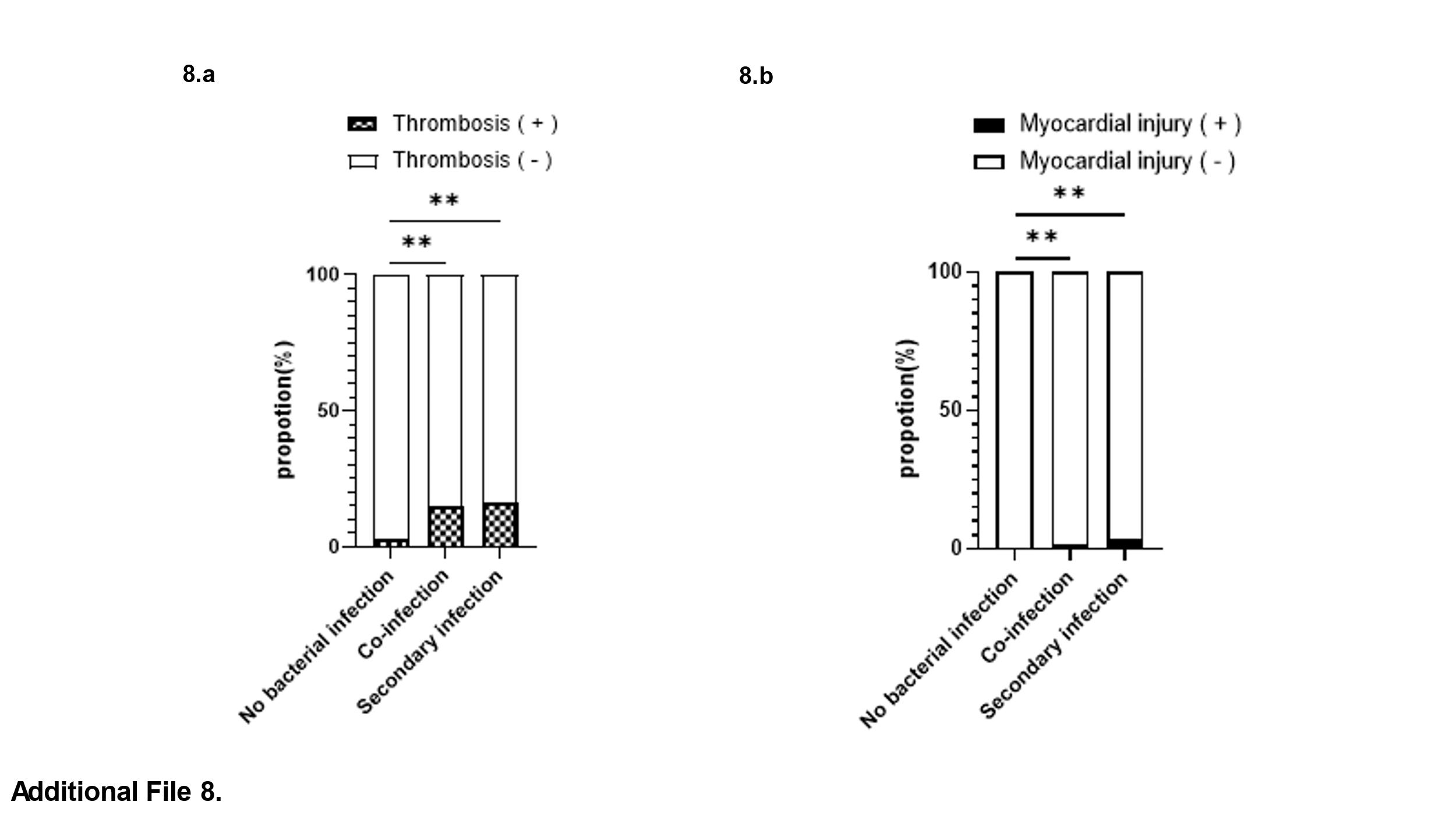


**Additional File 8. Proportion of thrombosis and myocardial injury in bacterial respiratory co-infection and secondary infection with coronavirus disease 2019 (COVID-19). a, b** Results of the univariate analysis for thrombosis and myocardial injury.

Proportions of thrombosis (a) and myocardial injury (b) in bacterial co-infection, secondary infection, and non-bacterial infection cases of coronavirus disease 2019 **(**COVID-19); **p < 0.01.
